# Artificial Intelligence in African Cardiovascular Care: Opportunities, Challenges, and Pathways to Improved Outcomes

**DOI:** 10.1002/puh2.70201

**Published:** 2026-02-18

**Authors:** Boluwatife Samuel Fatokun, Omosola Lydia Bolarin, Ahmed Muhammad Babandi, Pascal Mathew Okorobe, Chinwendu Janefrances Ezeagu, Ssentongo John, Hamzah Olaitan Muhammed, Obinna Joseph Mba

**Affiliations:** ^1^ Faculty of Basic Medical Sciences Kwara State University Malete Nigeria; ^2^ Department of Biochemistry Southwestern University Nigeria Okun‐Owa Ogun State Nigeria; ^3^ Department of Human Anatomy Federal University Dutsin‐Ma Dutsin‐Ma, Katsina State Nigeria; ^4^ School of Medicine Makerere University Kampala Uganda; ^5^ Department of Medical Laboratory Science Nnamdi Azikiwe University Awka Nigeria; ^6^ Department of Physiotherapy Mbarara University of Science and Technology Mbarara Uganda; ^7^ Department of Medical Laboratory Science Kwara State University Malete Nigeria; ^8^ Department of Pharmacology and Toxicology David Umahi Federal University of Health Sciences Uburu Ebonyi Nigeria

**Keywords:** Africa, artificial intelligence, cardiovascular disease, precision medicine, telemedicine

## Abstract

Cardiovascular disease (CVD) remains a leading cause of morbidity and mortality in Africa, accounting for over 1 million deaths annually. As CVD prevalence rises, Africa faces challenges in prevention, diagnosis, and management. Addressing this crisis requires innovative approaches, and artificial intelligence (AI) has emerged as a transformative solution. Studies already show how machine learning (ML) algorithms can predict various CVDs from patients’ data with accuracy of 73.8%–97.7%. This review explores the potential of AI to improve African cardiovascular care while discussing opportunities, challenges, and pathways for effective implementation. Hence, a comprehensive literature review was conducted using PubMed/MEDLINE, Google Scholar, Africa Journals Online (AJOL), and other online publications and grey literature relevant to the topic. This study discusses opportunities offered by AI to revolutionize cardiovascular care and improve diagnostic accuracy to include predictive analytics, ML, and telemedicine to process structured and unstructured data from m‐Health applications, wearable devices, and hospital records. Moreover, advanced applications could include genome‐wide association studies (GWAS) and precision medicine. Despite its advantages, AI integration faces challenges, including inadequate infrastructure, high implementation costs, policy and funding constraints, as well as limited digital literacy among healthcare providers. Data privacy concerns also remain critical, with only 36 of 55 African countries enacting data protection laws. Pathways to overcome these barriers include Africa's development of ethical standards for data use, investment in workforce training, collaborative partnerships, better funding structure, and strengthening of healthcare infrastructure and research.

## Graphical abstract

Artificial intelligence can transform cardiovascular care in Africa by improving early diagnosis, enabling personalized treatments, expanding telemedicine use, and improving rural healthcare access. Integrating AI‐driven tools with supportive policies, collaborative frameworks, training, funding, and infrastructure can overcome existing challenges and enhance African cardiovascular care.

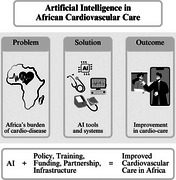

**Abbreviations**
CHWs: community healthcare workers
CVDs: cardiovascular diseases
DPIAs: data protection impact assessments
ECG: electrocardiogram
GWAS: genome‐wide association studies
ICT: Information and Communication Technology
I‐DAIR: International Digital Health & AI Research Collaborative
ITU: International Telecommunication Union
LMICs: low‐ and middle‐income countries
LVSD: left ventricular systolic dysfunction
ML: machine learning
NCDs: non‐communicable diseases
NDPR: Nigeria Data Protection Regulation
POPIA: Protection of Personal Information Act
PRS: polygenic risk score
RPM: remote patient monitoring
SSA: Sub‐Saharan Africa
WEF: World Economic Forum
WHO: World Health Organization

## 1 Background

Cardiovascular diseases (CVDs) account for almost one‐third of deaths globally and remain a significant public health challenge in Africa [1]. Data from Sub‐Saharan African (SSA) countries show that CVDs are responsible for approximately 13% of all deaths and 37% of all non‐communicable diseases (NCDs) deaths [2]. Moreover, an analysis of the Global Burden of Disease Study for SSA reported that although age‐standardized CVD deaths per 100,000 individuals metric showed a 14.4% decline, CVD raw counts in SSA shot up by 131.7% [3]. This is notwithstanding the issues of underreporting of these cases, poor systems of collecting epidemiology data, and shaky healthcare systems [2].

Africa's challenges in the management of CVDs cannot be overemphasized, despite its recognizable advancements in healthcare over the past years. A major part of this challenge is limited access to specialized care, resulting in delayed diagnosis and treatment [4]. As the majority of low‐ and middle‐income countries (LMICs) have not attained seamless integration of primary healthcare into their service delivery models, diagnosis and treatment are delayed [4]. Moreover, inadequacy or outright lack of healthcare infrastructures, like hospitals, clinics, medical equipment, and transportation options, has restricted access to healthcare services [5].

With the rise of artificial intelligence (AI), predictive analytics offers a promising way to improve patient outcomes and increase prognostic accuracy by leveraging advanced algorithms and machine learning (ML) techniques to analyze large amounts of patient data [6]. Clinicians can receive real‐time recommendations from AI‐powered decision support systems to help with diagnosis and treatment choices for various CVDs [7]. A study published on Cureus Journal of Medical Science already shows how ML algorithms predicted CVDs, such as acute coronary syndrome, atrial fibrillation, atrial septal defect, hypertension, and other cardiomyopathies, with accuracy ranging from 73.8% to 97.7% [8]. These developments represent a paradigm shift that can be leveraged for cardiovascular care in Africa; however, major obstacles like the absence of high‐quality medical data for AI algorithm training and worries about data security and privacy have been identified in AI implementation [9].

The subject of AI in African cardiovascular care has previously been accessed by a few studies; however, our article distinguishes itself by offering a comprehensive assessment and a clear implementation roadmap for AI to improve African cardiovascular care. Moreover, although adequately synthesizing peer‐reviewed literature for this study, this review also delves into grey literature, national programs, pilots, and industry deployments to produce the most complete picture of activities and frontiers on this subject, across Africa and beyond.

Specifically, this study highlights how AI can support early detection, improve diagnostic precision, enable personalized treatment, and strengthen patient monitoring. Current challenges, such as inadequate infrastructure, limited technical expertise, high costs, and ethical concerns around data use, were also examined. In addition, lessons from existing applications and frameworks on the subject of data protection, AI implementation, funding, training, partnerships, and policy creation in cardiovascular care in African countries and across the world were also considered.

## 2 Methodology

This literature review was performed using electronic search databases comprising PubMed/MEDLINE, Google Scholar, and Africa Journals Online (AJOL). Additional sources included grey literature from World Health Organization (WHO), African Union (AU), United Nations (UN), and other online publications relevant to the topic. A comprehensive literature search using keywords and associated Boolean operators related to “AI,” “CVDs,” “Africa,” “precision medicine,” and “telemedicine” was conducted to identify relevant studies exploring AI in African cardiovascular care.

Inclusion criteria comprised peer‐reviewed research articles and grey literature obtained from scientific online repositories that examined the challenges, opportunities, and pathways to improved outcomes in relation to AI in African cardiovascular care. Exclusion criteria comprised non‐peer‐reviewed articles and articles not addressing AI in African cardiovascular care in terms of opportunities, challenges, or policy. The selection process involved an initial screening of titles and abstracts to identify relevant articles, followed by a full‐text review to confirm eligibility in line with the selection criteria.

## 3 Opportunities for AI in African Cardiovascular Care

AI tools in medicine have evolved over the years. Historical milestones include Ledley and Lusted's probabilistic models for medical diagnosis in 1959 and Edward Shortliffe's expert system for infectious disease treatment and antibiotic selection in the 1970s called MYCIN [10, 11]. Moreover, the Internist‐I system in the 1970s was developed by the University of Pittsburgh's to harness AI to scale diagnostic support to over 500 conditions across internal medicine [12]. These efforts laid the foundation for modern applications of AI in healthcare. In recent years, AI tools in medicine, and particularly in cardiovascular care, have evolved from these early expert systems to electrocardiogram (ECG) recorders that can detect arrhythmias and algorithms that predict myocardial infarction (MI) from non‐enhanced magnetic resonance imaging (MRI) [13]. Moreover, AI‐assisted echocardiography that is enabled for automated chamber segmentation and ejection fraction estimation can significantly reduce operator dependence in facilities where trained sonographers or cardiologists are limited [14].

**FIGURE 1:**
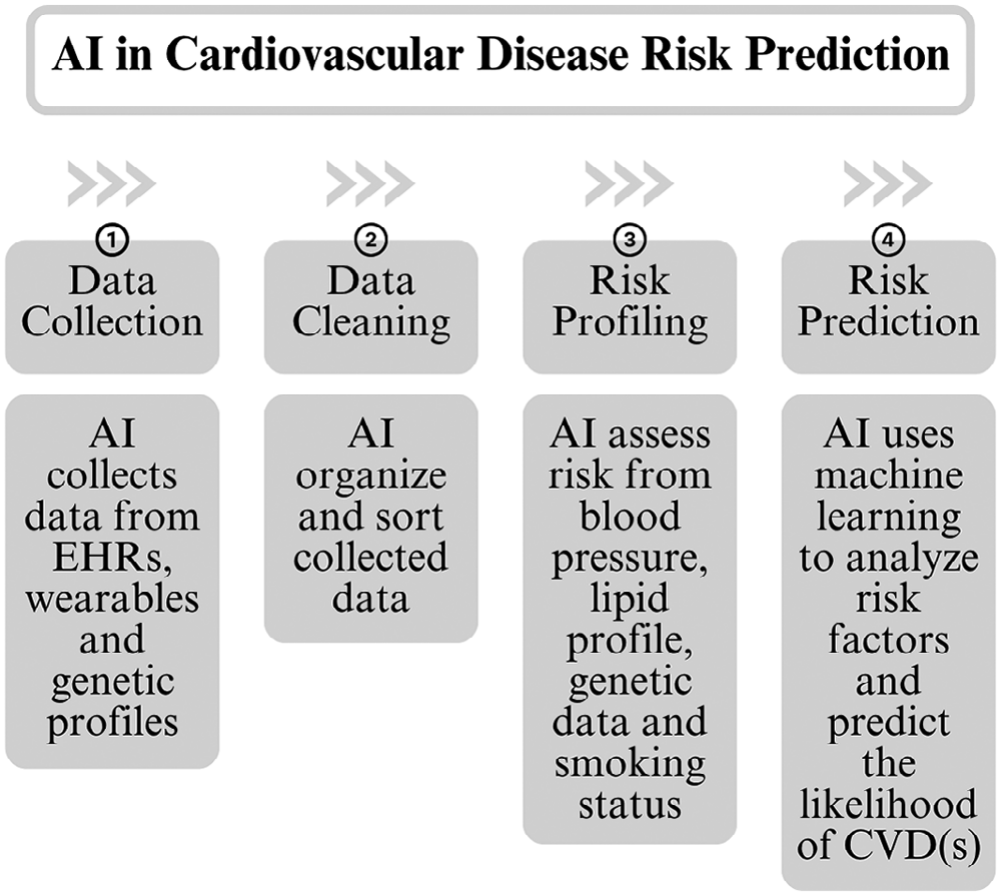
AI in cardiovascular disease risk prediction [13, 15, 18]. AI, artificial intelligence; CVD, cardiovascular disease.

AI‐driven telemedicine and remote patient monitoring (RPM) are another key area of interest [15]. With wearable technology, AI makes it possible to continuously monitor health parameters like blood pressure, heart rate, and glucose levels [15]. This helps in predicting possible health hazards, initiating early treatments, and modifying treatment programs for better health outcomes [15]. AI algorithms can also detect non‐linear relationships among risk factors, such as cholesterol levels, blood pressure, and genetic markers, leading to more precise predictions of CVD outcomes [13]. As illustrated in Figure 1, AI‐driven risk prediction models can integrate multiple data streams to provide a holistic view of patient risk. Moreover, AI does not simply replicate existing tools but can engage its risk profiling and predictive capacity to drive early intervention and personalized treatment. Additionally, AI infusion can help lower healthcare costs, promote inclusive healthcare, and enhance access to care in underserved areas [15].

Moreover, AI algorithms can improve internal organ image quality by reducing noise and enhancing detail, which is particularly valuable in cardiovascular imaging where precision is critical [16, 17]. AI tools can also streamline radiology workflows by automating processes like image segmentation and feature extraction, thereby saving time and improving efficiency for radiologists [16]. This results in clearer images that support more accurate diagnoses [16]. Studies have shown that radiographers in Africa are generally open to adopting AI technologies in medical imaging, recognizing their potential benefits in improving diagnostic accuracy, efficiency, and patient outcomes [17].

AI is also being integrated with several genetic tools to screen for CVDs and identify familial CVD risk factors peculiar to Africa [18]. One of such is genome‐wide association studies (GWAS), which is used to analyze large datasets and identify genetic loci linked to CVD [18]. AI algorithms can automate the detection of patterns and relationships in GWAS data, which may otherwise be too complex for traditional analysis [18]. This can accelerate the discovery of new genetic markers that may contribute to CVD susceptibility. AI can also enhance the accuracy and predictive power of polygenic risk scores (PRS) by refining the model with additional data, such as environmental and clinical factors. This enables a more personalized risk assessment and potential interventions before the onset of CVDs [18].

## 4 Challenges of Implementing AI in African Cardiovascular Care

The African healthcare sector faces several challenges, including the lack of training in AI technologies among healthcare professionals [19, 20]. This deficiency represents a major barrier to the effective implementation of AI for better healthcare outcomes [19]. The situation is amplified by the minimal availability of AI experts within the field to offer the training in the first place, making it more difficult, even for willing healthcare professionals, to fully leverage AI technologies [9, 19]. Overworked healthcare providers also often lack the time necessary for the implementation or training of these AI technologies [21, 22].

Urban and rural healthcare facilities differ significantly from each other, especially in the aspect of access to technology [19]. The many disadvantages that have hit African rural areas include the limited availability of health practitioners, poor health infrastructure, and inadequate funding for the use of advanced medical technologies [9, 23]. Moreover, although there are ongoing efforts to increase penetration of mobile phones and internet connectivity across Africa to address some of these disparities, many of them are yet to generate significant success [24].

The adoption of AI technology and infrastructure in healthcare is also greatly challenged by the high costs of purchase, maintenance, and upgrades of the required high‐technology computers [9]. For healthcare access and use in LMICs, these barriers will further increase because of heavy reliance on international funding, as the substantial investment required is often beyond the reach of many healthcare systems [9]. Moreover, the lack of the expertise to manage AI systems is another big barrier in settings where the preliminary setup might be doable [20].

Moreover, numerous African nations find it very difficult to build well‐equipped laboratories that can carry out high‐throughput sequencing, an essential experiment in genomic research [24]. Although the impact of AI in cardiovascular genomics could significantly better health outcomes, the cost of sequencing machines, bioinformatics software, and associated technologies are issues that many institutions across Africa still struggle with [24]. Consequently, there are only a handful of well‐funded research centers in countries like South Africa and Kenya, leaving many researchers reliant on international collaborations or outsourcing their work [24].

**FIGURE 2:**
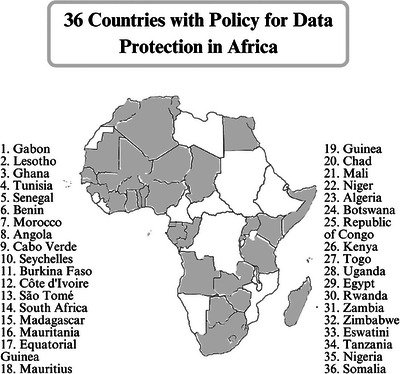
Thirty six countries with policy for data protection in Africa [25].

There are significant challenges regarding data privacy in digital health, especially concerning the ethical use of patient data for AI research [9, 20]. Ensuring the privacy and security of patient information is paramount, but the integration of AI technologies complicates these efforts [20]. Moreover, in resource‐limited settings like Africa, these challenges are further compounded by issues related to obtaining informed patient consent, enactment of data protection laws, the increasing risk of cyberattacks, and lack of legal clarity as regards large‐scale adoption [19, 21]. As illustrated in Figure 2, only 36 out of 55 African countries have enacted data protection laws as of January 2024. The gap highlighted here shows a major challenge, and it is one of the reasons for weak AI implementation in many African regions.

Table 1 anchors the challenges previously outlined in this section to corresponding, globally recognized indicators. Internet penetration variability across Africa was quantified by individuals using the internet percentages, as it directly determines feasibility of telemedicine, wearables, RPM, or any AI‐enabled cardiovascular care [26]. Cybersecurity vulnerability was assessed using the ITU (International Telecommunication Union) Global Cybersecurity Index of 2024 that reflects national capacity to secure telecommunications/Information and Communication Technology (ICT) networks, and by extension patient data and AI systems as well [27]. Availability of data protection laws was also recorded for every country with clear federal/national guidelines [28, 29]. Other stated challenges, including the general lack of funding, training, and expertise required for adoption, implementation, and maintenance of AI in Africa and African cardiovascular care, were evaluated using the digital skills score sourced from the World Economic Forum (WEF) Global Competitiveness Report of 2019 and the Government AI Readiness Index of 2024 [30, 31].

**TABLE 1:** Global snapshot of artificial intelligence (AI) implementation readiness by region.

Region	Country	Individuals using the Internet 2022 (% of the population) sourced from ITU [26]	Global Cybersecurity Index 2024 (Tiers 5–1) by ITU [27]	Federal/National Data Protection Laws (clearly defined) [28, 29]	Government AI Readiness Index 2024 (0–100) by Oxford Insights [30]	Digital skills among active population (1–7) sourced from WEF [31]
**Western Africa**	Nigeria	35.5	Tier 3	Nigeria Data Protection Act (NDP Act) of 2023	43.3	3.4
	Mali	33.1	Tier 4	Autorité de Protection des Données à Caractère Personnel (APDP) of 2013—Personal Data Protection Authority	32.3	3.6
**Southern Africa**	South Africa	74.7	Tier 2	Protection of Personal Information Act 4 (POPIA) of 2013	52.9	3.3
	Angola	39.3	Tier 4	Personal Data Protection Law No. 22/11 of 2011	26.9	2.5
**Northern Africa**	Egypt	72.2	Tier 1	Personal Data Protection Law No. 151 of 2020	55.6	4.7
	Sudan	28.7	Tier 4	—	—	—
**Eastern Africa**	Kenya	40.8	Tier 1	The Data Protection Act of 2019	43.6	4.6
	Ethiopia	19.4	Tier 3	—	38.3	3.8
**Central Africa**	Cameroon	43.9	Tier 3	—	33.5	3.9
	Democratic Republic of Congo	27.2	Tier 3	—	22.1	2.8
**Asia**	Nepal	49.6	Tier 3	—	33.1	3.7
	China	75.6	Tier 2	Personal Information Protection Law (PIPL) of 2021	72.0	4.7
**Latin America**	Mexico	78.6	Tier 2	Ley Federal de Protección de Datos Personales en Posesión de Particulares (LFPDPPP) of 2025—Federal Law on the Protection of Personal Data Held by Private Parties; Ley General de Protección de Datos Personales en Posesión de Sujetos Obligados (LGPDPSO) of 2017—The General Law for the Protection of Personal Data in Possession of Obligated Subjects	53.3	3.8
	Brazil	80.5	Tier 1	Lei Geral de Proteção de Dados (LGPD) of 2018—The Brazilian General Data Protection Law	65.9	3.1
**Europe**	United Kingdom	95.3	Tier 1	General Data Protection Regulation (UK GDPR) of 2021	78.9	4.9
	France	85.3	Tier 1	General Data Protection Regulation (EU GDPR 2016/679) of 2016	79.4	4.5
**North America**	United States of America	97.1	Tier 1	—	87.0	5.3
	Canada	94.0	Tier 2	Personal Information Protection and Electronic Documents Act (PIPEDA) of 2000	78.2	5.1

Abbreviations: ITU, International Telecommunication Union; WEF, World Economic Forum.

This snapshot reveals pronounced disparities within Africa and between Africa and other regions. Countries such as Egypt and South Africa demonstrate relatively consistent readiness, with stronger cybersecurity scores, clear data protection laws, and higher digital skills levels, reflecting deliberate national investment in digital governance (Table 1). Kenya and Nigeria stand out for the availability of comprehensive data protection laws but require more national commitment to digital skill acquisition and cybersecurity capacity (Table 1). The region of Central Africa is shown as the relatively least‐ready within Africa with low internet penetration, limited skills data, and absent governance frameworks (Table 1). Moreover, there is a striking variance in readiness on a country‐by‐country basis within the countries of Southern and Northern Africa (Table 1).

In contrast, global comparators, such as the United Kingdom, China, and Mexico, show higher digital skills scores and stronger cybersecurity capacity, as well as established and evolving national AI strategies and laws (Table 1). Notably, the United States scores highly on skills, government AI readiness, and cybersecurity but lacks a federal data protection law, reflecting a fragmented regulatory environment (Table 1). These contrasts illustrate both universal and region‐specific gaps but also highlight that Africa's AI readiness barriers are structural, interlinked, and consequential on adoption of AI in African cardiovascular care.

## 5 Case Studies and Success Stories of AI in African Cardiovascular Care

One of the cardiovascular medicine‐specific applications seen was an external validation analysis done in Uganda, where the Mayo Clinic AI tool detected left ventricular systolic dysfunction (LVSD) in hospital patients [32]. Another was a collaboration with Healthtech Mali in applying AI to enhance the diagnosis and treatment of cardiac conditions in Bamako, Mali [33]. This has improved the level of understanding of test results and promotes better decision‐making for treatment purposes [33]. Moreover, Life Bank, an online system for blood services, has been able to connect Nigerian hospitals to blood banks within the country, leading to better health outcomes for patients [33].

Another is the connection of an ECG signal, blood pressure, or pulse monitor through Bluetooth or a wearable device to a smartphone for data monitoring and analysis [34]. This is used to monitor a possible heart attack or failure with a responsive danger alarm in the patient through a signal that would be generated by the AI tool [34]. This has been applied in telemonitoring of cardiovascular risk factors such as high blood pressure, high cholesterol, and blood sugar; remote monitoring of high‐risk cardiovascular patients especially the geriatrics; diagnosis and prognosis of CVD [34].

Moreover, a clinical trial in Nigeria collated patients’ electronic medical records for the identification of pregnancy‐related heart problems and stored them in a data bank to give insights for better diagnosis, prognosis, and treatment of pregnancy‐related heart disease [23]. Moreover, the African School of Hypertension, a project of the African regional advisory group of the International Society of Hypertension, has been engaging community healthcare workers (CHWs) in the evaluation of AI usage amongst African healthcare institutions [23]. This has involved building smartphone AI applications that can link up CHWs with patients managing various CVDs [23].

Beyond these projects and tools, some African nations have already introduced data protection and governance measures that can serve as valuable models for others. South Africa's Protection of Personal Information Act (POPIA) of 2013 that was implemented in 2020 is one of the continent's most comprehensive laws, setting‐specific requirements for data processing and accountability [35]. In East Africa, Kenya's Data Protection Act of 2019 established an independent Data Protection Commissioner, with recent guidance notes outlining‐specific obligations for health institutions [36]. In West Africa, Nigeria has also moved from the earlier Nigeria Data Protection Regulation (NDPR) to a National Data Protection Act of 2023, hence establishing a dedicated enforcement agency and laying the groundwork for structured governance of personal data in healthcare [37].

At the continental level, the AU Malabo Convention on Cyber Security and Personal Data Protection provides a harmonized baseline for cross‐border data flows and cybersecurity cooperation, which is vital for multicountry AI research [38]. It was adopted in 2014, and implementation began in 2023 [38]. These frameworks, though unevenly implemented, provide concrete models that, if strengthened and adapted, could be instrumental in building the trust and governance structures needed to support AI integration in cardiovascular care.

## 6 Pathways to Improved AI Integration in African Cardiovascular Care

ML algorithms (supervised, unsupervised, and reinforcement learning models) should be leveraged by researchers to develop effective disease detection tools that can analyze large datasets and identify patterns indicative of CVDs [39]. In medical imaging, software, such as CaRi‐Heart, which uses AI to analyze computed tomography (CT) coronary angiography images and predicts the cardiac risk in patients with suspected coronary artery disease, should be developed [40]. Moreover, AI‐assisted electrocardiography should be leveraged, particularly in at‐risk populations to detect cardiovascular abnormalities and improve early stage diagnosis [41]. In genomics, the integration of AI in precision medicine also offers opportunities for personalized diagnosis and prognostication by combining genomic and non‐genomic determinants with patient information [42].

There should also be development of policies that clearly define the scope of AI applications in cardiovascular care, as they will foster trust and ensure that AI systems are used responsibly [43]. A comparative analysis between Nigeria's Data Protection Act of 2023 and Kenya's Data Protection Act of 2019 shows similarities in core privacy principles in data‐subject rights, data protection impact assessments (DPIAs), data protection officers (DPOs), breach notification, and restrictions on health/sensitive data [36, 37]. However, these acts can be further strengthened by establishing clear guidelines as regards the use of personal data for ML algorithms, the requirement of AI DPIAs to document model inputs (e.g., ECG reports, images, and genomics), risk of bias, validation metrics, and ensure there are human safeguards (e.g., clinician oversight in the use of AI for cardiovascular health).

Partnerships between public and private setups, NGOs, and global AI institutions also offer opportunities to pool resources for innovation, infrastructure building, and adoption of AI technologies [44, 45]. These efforts in cooperation could improve access to AI solutions in cardiovascular care and subsequently lead to the development of sustainable health systems for the diverse populace [46]. Ongoing initiatives, such as the WHO International Digital Health & AI Research Collaborative (I‐DAIR) collaboration and the Artificial Intelligence for Development (AI4D) HealthAI project, demonstrate that Africa is already participating in regional and international networks to advance AI governance and health innovation [47, 48]. Building on and expanding these collaborations will be essential to ensure harmonized approaches across the continent.

African institutions who are actively embedding AI into educational curricula and are making strategic investments into AI‐focused research centers should also be encouraged, whereas room should be created for more [49]. Projects like that of Carnegie Mellon University Africa that intends to develop AI‐assisted stethoscopes that can accurately process patient data and assess CVD severity would ensure exponential growth in African cardiovascular care [50]. Moreover, Uganda's AI Health Lab at Makerere University aim of revolutionizing diagnostics, treatment plans, and personalized care should be encouraged [51]. As AI‐enhanced tools and applications become more available and accessible, nonspecialists, such as CHWs, would be capable of effectively better navigating CVDs and improving healthcare services [52].

Mobile health (mHealth) applications are transforming cardiovascular care by integrating AI to enhance diagnostics, monitoring, and patient management [53]. To maximize their impact in Africa, mHealth apps should focus on patient engagement and accessibility by delivering educational content and actionable health insights directly to users [53]. African countries should also leverage telecom partnerships that enhance network coverage, make data usage more affordable, and increase the accessibility of health applications for better cardiovascular health outcomes, such as seen with Safaricom for the M‐TIBA app in Kenya [54].

Others include the development of AI‐enhanced sensors as enabling technologies for next‐generation healthcare and biomedical platforms, which are area that can enable the successful integration of AI in CVD care in Africa [55]. Models, such as CardioRiskNet, a hybrid AI‐based model designed for explainable risk prediction and prognosis in CVD, are intriguing AI model that can be sustainably implemented for African cardiovascular care [56]. Sustaining and expanding programs that veered toward building local expertise, such as seen with the efforts of the Africa‐based AI for Public Health initiative, will be vital for developing the skilled workforce required to drive AI adoption in cardiovascular care [57].

**FIGURE 3:**
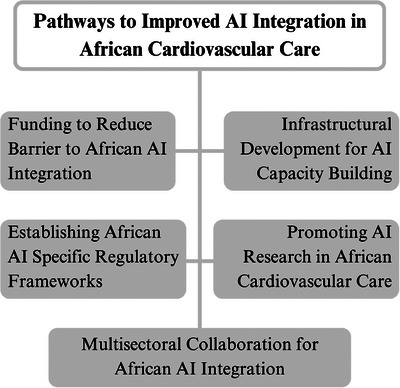
Pathways to improved AI integration in African cardiovascular care [28, 31, 34]. AI, artificial intelligence.

Figure 3 summarizes the pathways for AI integration in African cardiovascular care as discussed earlier and highlights their interdependence as a coordinated approach across multiple domains is required for effective integration. The highlighted pathways include supportive policies, investment in infrastructure, workforce training, research, and collaborative partnerships. In summary, Table 2 fosters an integrated understanding of how previously identified opportunities intersect with existing constraints and provides feasible and strategic solutions necessary to support integration and adoption of AI opportunities in African cardiovascular care.

**TABLE 2:** Summary of artificial intelligence (AI) opportunities, barriers, and implementation pathways for African cardiovascular care.

AI opportunity	Corresponding barrier	Implementation pathway
AI‐enhanced ECG for early detection of LVSD and arrhythmias [13]	Shortage of cardiologists and limited training of medical personnel for AI use [9, 19, 23]	Infusion of AI into medical curricula and development of CHW‐focused AI training modules for rural access [49, 52]
AI‐assisted echocardiography (automated chamber segmentation and ejection fraction estimation) [14]	Few echo‐trained clinicians, varying availability of equipment, and high cost of AI‐echo software [9, 23]	Funding for infrastructural support, regional collaboration to foster availability of equipment and training for clinicians [44, 45]
AI‐supported imaging (CT and MRI) and improved image quality [16]	Shortage of radiologists, varying availability of imaging suites across the African region, high infrastructure cost [9, 23]	Regional hubs and cloud‐based reading with data protection and clinical oversight safeguards
Cardiovascular genomics and precision cardiology [18]	Limited African genomic datasets, weak bioinformatics capacity [24]	Creation of national biobanks, partnerships that scale local bioinformatics training across Africa [42, 45]
Wearables and remote patient monitoring for hypertension and other cardiomyopathies [15]	Low rural internet penetration, relative cost of implementation [22, 23]	Telecom partnerships, low‐bandwidth data transmission, and provision of subsidies [45, 54]
Population‐level risk prediction (AI‐risk scores) [13]	Fragmented and varying data availability, portability issues, and weak data governance [19, 21]	Implementation of data laws, strengthened enforcement, and creation of national cardiovascular data registries [43]

Abbreviations: CHW, community healthcare worker; CT, computed tomography; ECG, electrocardiogram; LVSD, left ventricular systolic dysfunction; MRI, magnetic resonance imaging.

## 7 Conclusion

The rise of AI in African cardiovascular care has shown potential for improved diagnosis, personalized treatment, and remote monitoring of patients. Through AI‐driven solutions, such as ML, wearable technologies, m‐Health, and genomics, healthcare systems in Africa can address critical gaps in the current systems of cardiovascular care, particularly in underserved regions. These AI opportunities highlight the possibility of reducing morbidity and mortality associated with CVDs while fostering better healthcare access across the continent.

However, significant barriers are hindering the implementation of AI in African health systems, particularly in cardiovascular care. These issues include insufficient healthcare infrastructure, high cost of implementation, limited technical expertise, and training as well as ethical and policy challenges. Thus, for AI to be effectively integrated in Africa cardiovascular care, swift action should be taken to integrate AI in medical curricula, develop robust policies for responsible AI use, establish regional AI‐health data centers, support African‐based institutions and initiatives, as well as promote equitable cross‐border AI collaboration.

## Author Contributions

Boluwatife Samuel Fatokun was responsible for conceptualization, project administration, creation of figures, as well as the review and preparation of the first and the final draft. Omosola Lydia Bolarin, Ahmed Muhammad Babandi, Pascal Mathew Okorobe, Chinwendu Janefrances Ezeagu, Ssentongo John, and Hamzah Olaitan Muhammed were responsible for data collection and initial manuscript writing. Obinna Joseph Mba was responsible for review and preparation of the final draft.

## Funding

The authors have nothing to report.

## Ethics Statement

The authors have nothing to report.

## Consent

The authors have nothing to report.

## Conflicts of Interest

The authors declare no conflicts of interest.

## Data Availability

Data sharing is not applicable to this article as no datasets were generated or analyzed during the current study.
